# Redox Regulation of Monodehydroascorbate Reductase by Thioredoxin y in Plastids Revealed in the Context of Water Stress

**DOI:** 10.3390/antiox7120183

**Published:** 2018-12-06

**Authors:** Hélène Vanacker, Marjorie Guichard, Anne-Sophie Bohrer, Emmanuelle Issakidis-Bourguet

**Affiliations:** Institute of Plant Sciences Paris-Saclay (IPS2), UMR Université Paris Sud—CNRS 9213—INRA 1403, Bât. 630, 91405 Orsay CEDEX, France; helene.vanacker@u-psud.fr (H.V.); marjorie.guichard@cos.uni-heidelberg.de (M.G.); bohreras@msu.edu (A.-S.B.)

**Keywords:** thioredoxin, monodehydroascorbate reductase, water stress, protein oxidation, antioxidants, ascorbate, glutathione

## Abstract

Thioredoxins (TRXs) are key players within the complex response network of plants to environmental constraints. Here, the physiological implication of the plastidial y-type TRXs in Arabidopsis drought tolerance was examined. We previously showed that TRXs y1 and y2 have antioxidant functions, and here, the corresponding single and double mutant plants were studied in the context of water deprivation. TRX y mutant plants showed reduced stress tolerance in comparison with wild-type (WT) plants that correlated with an increase in their global protein oxidation levels. Furthermore, at the level of the main antioxidant metabolites, while glutathione pool size and redox state were similarly affected by drought stress in WT and *trxy1y2* plants, ascorbate (AsA) became more quickly and strongly oxidized in mutant leaves. Monodehydroascorbate (MDA) is the primary product of AsA oxidation and NAD(P)H-MDA reductase (MDHAR) ensures its reduction. We found that the extractable leaf NADPH-dependent MDHAR activity was strongly activated by TRX y2. Moreover, activity of recombinant plastid Arabidopsis MDHAR isoform (MDHAR6) was specifically increased by reduced TRX y, and not by other plastidial TRXs. Overall, these results reveal a new function for y-type TRXs and highlight their role as major antioxidants in plastids and their importance in plant stress tolerance.

## 1. Introduction

As sessile organisms plants are continuously exposed to environmental fluctuations. In order to maintain photosynthetic carbon fixation efficiency, especially in varying light conditions, they have evolved diverse adaptive strategies including redox regulation. Indeed, thiol-based redox systems, i.e., glutathione and thioredoxins (TRXs), play major roles in the complex redox regulatory network underlying plant responses to fluctuating environmental cues.

TRXs are small ubiquitous redox proteins catalyzing dithiol–disulfide exchange reactions with their target enzymes thanks to the presence of 2 reactive Cys residues in the conserved WC(G/P)PC motif in their active site. TRXs can fulfill two types of functions, either as redox regulators that usually allow the reductive activation of their target enzymes, or as reducing substrates that provide reducing power for antioxidant systems that detoxify H_2_O_2_ [1].

Plant genome sequencing data revealed that photosynthetic organisms possess a high number of trx genes including numerous isoforms localized in chloroplasts that were classified into five subtypes. In Arabidopsis (*Arabidopsis thaliana*) 10 plastidial TRXs were found: two TRXf, four TRX m, one TRX x, two TRX y, and one TRX z [2,3]. Biochemical studies enabled functional specificity to be assigned to different plastidial TRXs, leading to a global picture in which the f and m-type TRXs are regulators of enzymes directly or indirectly linked to photosynthetic carbon metabolism, while the x and the y types appear to have antioxidant functions [4,5,6,7]. TRX z which displays unique properties among plastidial TRXs [8,9] has been recently validated as a regulator of plastidial gene expression [10]. Recent studies, using Trx mutant plants and over-expressors, allowed some of these functions to be confirmed *in planta*, enabling evaluation of the degree of redundancy between the various TRX types. Most of these studies have investigated the roles of TRXs f and TRXs m in the regulation of stromal enzymes to adjust their activity to varying photosynthetic electron flow under fluctuating light intensities [11,12,13,14]. Moreover, Arabidopsis TRXs y1 and y2 were shown to play antioxidant roles whatever their redox state, by serving as reducing substrates [5,6,7,15], performing oxidative activation of G6PDH [16], and maintaining leaf MSR (Methionine Sulfoxide Reductase) capacity in high light conditions [17].

As a first approach to identify TRX y protein partners, we previously performed a proteomic study of putative targets of y-type TRX in Arabidopsis roots, since Trx y1 is mostly expressed in non-photosynthetic organs [5,9,18]. A monocysteinic mutant of TRX y was used as a bait to trap protein partners by affinity chromatography in a root crude extract. Seventy-two proteins have been identified, functioning mainly in metabolism, detoxification and response to stress, as well as in protein processing and signal transduction. In particular, we identified the plastidial monodehydroascorbate reductase (MDHAR) as a TRX-linked protein [19]. This enzyme is considered to play a role in ROS detoxification, being part of the ascorbate-glutathione pathway using ascorbate (AsA) as reducing substrate. AsA oxidation leads to monodehydroascorbate which can be recycled back to AsA thanks to MDHAR using NAD(P)H as the reductant [20]. Therefore, MDHARs play an important role in the response of plants to oxidative stress by maintaining the intracellular ascorbate redox state mainly in the reduced state.

Drought is one of the most serious environmental stresses affecting plant performance and crop yield and is expected to become more widespread and severe due to climate change [21]. Moreover, it is well established that cell redox homeostasis is disturbed under dehydration stress [22]. In this context, we studied the impact of the TRXs y mutations on the antioxidant response of Arabidopsis plants challenged with drought stress.

## 2. Materials and Methods

### 2.1. Reagents

All biochemical reagents were purchased from Sigma-Aldrich (Sigma-Aldrich Chimie, Saint-Quentin Fallavier, France), unless otherwise mentioned.

### 2.2. Plant Material

All the Arabidopsis (*Arabidopsis thaliana*) mutants used in this study were in the Columbia (Col-0) genetic background. Knock-out plants (T-DNA insertion mutants) in the trx y1 (At1g76760) or/and trx y2 (At1g43560) gene(s), either single (*trxy1-1* and *trxy1-2*, two allelic mutants lines, and *trxy2*) or double (*trxy1y2* obtained from *trxy1-2 and trxy2*) mutant lines, used in this study were previously obtained and described [17].

### 2.3. Plant Growth Conditions and Water Stress Treatment

15-days old seedlings (obtained under in vitro short day conditions i.e., 8-h photoperiod at 100 µmol photons m^−2^ s^−1^ for 8 h, 20 °C/18 °C (day/night) temperature regime and a relative humidity of 65%, on ½ MS agar medium) were individually transferred into a ready-to-use plant multiplication plug system (Fertiss 455.40, FERTIL, Boulogne-Billancourt, France) and further grown in a controlled-environment growth chamber under an 8-h photoperiod at an irradiance of 150 µmol photons m^−2^ s^−1^. The temperature regime was 20 °C/18 °C (day/night) and the relative humidity was 65%. Plants were irrigated twice a week with fertilizing solution (NPK 14.12.32, PLANT-PROD, FERTIL). Plants were grown for 3 weeks and either sampled or further cultivated in control or drought stress conditions and sampled for experiments as indicated. Samples were rapidly frozen in liquid nitrogen and stored at −80 °C until analysis. All data are means ± SD of at least three leaf samples obtained from different plants, and experiments were repeated at least twice. Drought stress was imposed by stopping irrigation of 3-week-old plants.

### 2.4. Relative Water Content Measurement

Relative water content was calculated according to the equation of RWC (%) = [(FW − DW)/(TW − DW)] × 100, by measuring fresh weight of excised leaves (FW); turgid weight after dipping in water for 4 h (TW), and dry weight after overnight drying at 80 °C (DW).

### 2.5. mBBR Labelling and Quantification

The monobromobimane (mBBr) probe was used for detection of reduced proteins since it fluoresces following its covalent interaction with thiols (reduced form of Cys). Leaf samples (100 mg) were ground in Tris-HCl 100 mM pH 7.6 supplemented with the broadly used cocktail of protease inhibitors (special plant from Sigma-Aldrich P9599). Free thiols were directly labeled with 2 mM mBBr included in the extraction buffer (30 min incubation at room temperature). After centrifugation (14,000× *g*, 10 min, at 4 °C), soluble proteins were quantified (Qubit protein assay kit, Life technologies, Thermo Fisher Scientific, Illkirch, France) and resolved by SDS-PAGE in 4–20% acrylamide gels (25 µg protein sample loaded per well). Reduced proteins were visualized under UV before staining of total proteins with Coomassie blue. The mBBr fluorescence and Coomassie colorimetric signals were quantified using the VisionCapt software (Quantum ST5 from Vilber-Lourmat, Vilber, Marne-La-Vallée, France).

### 2.6. Protein Carbonylation

The spectrophotometric dinitrophenyl hydrazine (DNPH) method was used for the determination of carbonyl groups in proteins. Centrifugation-clarified leaf samples were prepared as described above (without mBBr) before removal of nucleic acids by precipitation with streptomycin sulphate 1% (*w*/*v*) (20 min incubation and centrifugation at 12,000× *g* at room temperature). Supernatants were mixed with 7.5 mM DNPH final concentration and incubated for 15 min prior to precipitation in presence of TCA 10% (*v*/*v*). The pellets were washed five times with ethanol:ethylacetate (1:1), dried and finally dissolved in 6 M guanidine hydrochloride and the absorption at 370 nm was measured. Carbonyl content was calculated using a molar absorption coefficient for aliphatic hydrazones of 22,000 M^−1^ cm^−1^ and protein recovery was estimated by measuring the A276 and corrected using the formula [Protein] = (*A*276 − 0.43 × *A*370) established previously [23].

### 2.7. MDHAR Activity Measurements

For measurement of MDHAR (EC 1.6.5.4) activity in leaves, freshly harvested leaf tissue (in the middle of the light period, 250 mg) was extracted in 1 mL of 50 mM MES-KOH buffer (pH 6.0), containing 40 mM KCl, 2 mM CaCl_2_, and 1 mM L-ascorbic acid (AsA, freshly prepared). The homogenate was centrifuged at 14,000× *g* for 10 min at 4 °C, and the supernatant analyzed immediately for MDHAR activity.

MDHAR activity in leaf extracts was assayed spectrophotometrically at 25 °C by the slightly modified method described previously [24]. The MDHAR reaction was started by adding 0.4 unit of ascorbate oxidase (1 unit defined as the amount of enzyme catalyzing the oxidation of 1 µmol ascorbate per min) to generate the monodehydroascorbate radical in the reaction mixture (1 mL) containing 50 mM HEPES-KOH buffer (pH 7.6), 2.5 mM AsA, 0.25 mM NADPH and 15 µM FAD. The activity was determined by following for 2 min the decrease in absorbance at 340 nm due to the oxidation of NADPH using an extinction coefficient of 6.22 mM^−1^ cm^−1^. The same protocol was used to measure the MDHAR activity of recombinant protein (obtained as described below) following 15 min incubation in 100 mM Tris-HCl pH 7.9 at room temperature (1 µM MDHAR6 in the final 1 mL cuvette assay) in the presence or absence of 10 mM dithiothreitol (DTT), alone or with 10 μM TRX.

### 2.8. Production and Purification of Recombinant Proteins

The cDNA sequence corresponding to the MDHAR6 (At1g63940) was obtained from the “Arabidopsis Biological Resource Center” (ABRC, DKLAT1G63940; clone U09541) and amplified by PCR (primers used detailed in Supplemental Table S1) and cloned at *Nco*I and *Xho*I restriction sites into the pGENI vector [9] allowing the production of AtMDHAR6 (without transit peptide, starting at Phe39) with a Strep-tag at its C-terminus in BL21 (DE3) *E. coli* cells. Bacteria were cultured, at 37 °C, in Luria-Bertani broth (LB) medium supplemented with 100 µg/mL ampicillin. AtMDHAR6 protein production was induced with 500 µM isopropyl-*β*-d-thiogalactopyranoside (IPTG) for 3 h at 37 °C. Cells were harvested by centrifugation at 8000× *g* for 20 min at 4 °C, resuspended in 30 mM Tris-HCl, pH 7.9 with a cocktail of protease inhibitors (Complete EDTA-free protease inhibitor cocktail, Roche Diagnostics), disrupted by three passages through a French press (10,000 p.s.i.) and soluble extract was cleared by centrifugation at 19,000× *g*, 4 °C for 45 min. The supernatant was loaded onto a *Strep*-Tactin affinity column (*Strep*-Tactin^®^ Sepharose^®^, IBA GmbH Göttingen, Germany), pre-equilibrated with buffer 100 mM Tris-HCl, pH 8.0, 150 mM NaCl, 1 mM EDTA. After washing with the same buffer, the recombinant protein was eluted with 2.5 mM desthiobiotin and dialysed against 30 mM Tris-HCl, pH 7.9, 1 mM EDTA. Purity and molecular mass of the protein were checked by SDS-PAGE with Coomassie blue staining. Protein concentrations were determined spectrophotometrically at 450 nm, corresponding to the flavin adenine nucleotide (FAD) absorption peak, using a molar extinction coefficient of 11.3 mM^−1^ cm^−1^ [25]. The identity and purity of recombinant AtMDHAR6 protein preparation was confirmed by mass spectrometry. Recombinant Arabidopsis plastidial TRXs (TRX f1, TRX m1, TRX x and TRX y2) were obtained and purified as previously described [4,5].

### 2.9. Determination of Ascorbate and Glutathione

Antioxidant metabolites were extracted from whole leaves as described previously [26]. The content of AsA and DHA were measured as described previously via the decrease in *A*265 after the addition of AsA oxidase [27]. DHA content was calculated as the difference between total and reduced AsA. Total AsA was measured after incubation of the sample with DTT (2.4 mM) for 15 min. Total glutathione (GSH and GSSG) and GSSG were measured as described previously [28]. Total glutathione was estimated via the increase in *A*412 after the addition of Glutathione Reductase (GR) and NADPH.

### 2.10. Statistical Analysis

All analyses were performed according to a completely randomized design. Each experiment was repeated 2–4 times. The results were expressed as means and error bars were used to show standard deviation (±SD). Significant differences between genotypes (WT vs. mutant) or growth conditions (control vs. stress) were compared using Student’s *t*-test, with *p* < 0.05 considered as significantly different.

## 3. Results

### 3.1. TRX y1 and TRX y2 are Important for Arabidopsis Drought Stress Tolerance

**Figure 1:**
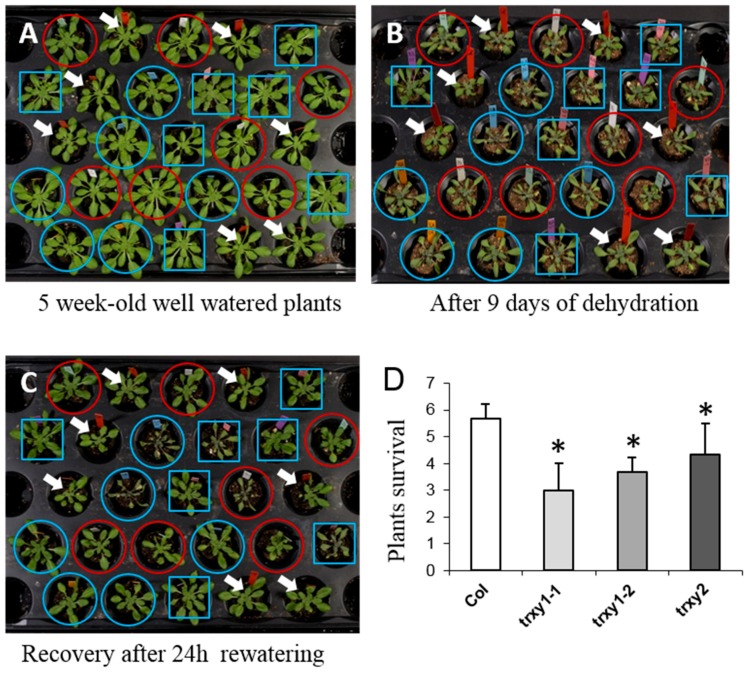
Water stress tolerance of WT and *trxy* mutant plants. After 3 weeks of growth under (**A**) standard conditions; (**B**) plants watering was stopped for 9 days and then (**C**) rehydrated for 24 h before (**D**) plant survival was monitored. Data correspond to means ± SD (*n* = 6). The asterisk (*) indicates a mutant sample significantly different from wild-type (*p* < 0.05). White arrows, blue circles, blue squares and red circles indicate wild-type (Col), *trxy1-1*, *trxy1-2* and *trxy2* plants, respectively.

Previous work had suggested that both TRXs of the y-type could play an important role in determining tolerance of Arabidopsis plants to environmental stress [17]. Here, we studied the impact of the TRXs y mutations on the antioxidant response after drought stress. We first studied the tolerance of TRXs y1 and y2 single mutants to dehydration (two allelic mutant lines *trxy1-1* and *trxy1-2* and one *trxy2* mutant line). While in control growth conditions, *trxy1* and *trxy2* mutant plants developed similarly to wild-type (WT) plants (Figure 1A), they showed an increased sensitivity to dehydration as evidenced by their wilted phenotype (Figure 1B). This behavior was confirmed by the lower capacity of mutant plants to recover from a 9-day period of water deprivation (Figure 1C,D). Indeed, 24 h after re-watering, while almost all WT plants were still alive, only 50–60% of the *trxy1* and *trxy2* mutant plants were able to recover from the stress.

**Figure 2:**
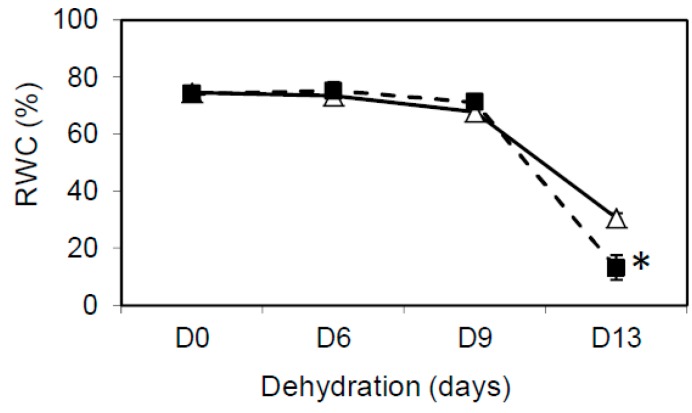
Effect of water stress on the relative water content (RWC, in %) in Col-0 (open triangles) and the double mutant *trxy1y2* (dark squares). Means ± SD are the average of 6 biological repeats from 2 independent experiments (*n* = 6). The asterisk (*) indicates a mutant sample significantly different from WT (*p* < 0.05).

These preliminary results provided a first indication of a functional role for TRX y1 and TRX y2 in determining the tolerance of Arabidopsis to water deficiency as well as possible redundancy between the two y-type TRX isoforms in this stress context. To further investigate this question, we obtained a *trxy1y2* double mutant line by crossing the *trxy1-2* and *trxy2* single mutants. The *trxy1y2* double mutant did not show any obvious phenotype in optimal growth conditions ([17], and this work). In the double mutant, leaf relative water content (RWC), remained high (ca. 80%) during 9 days of dehydration, but subsequently decreased more markedly than in the WT (Figure 2). Thus, y-type TRXs seemed to be important for the tolerance of Arabidopsis to water deficiency, suggesting an impaired capacity of the corresponding double mutant *trxy1y2* to cope with stress-triggered oxidative effects at the molecular level.

### 3.2. Global Protein Oxidation is Enhanced in the trxy1y2 Mutant Under Drought Stress

**Figure 3:**
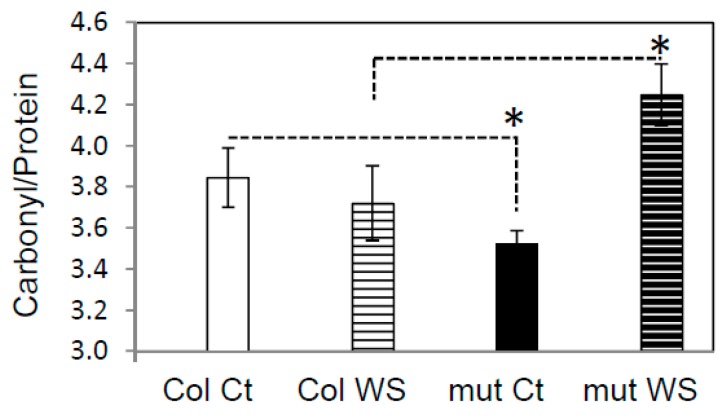
Protein carbonylation levels in wild-type (WT) and *trxy1y2* mutant leaves after 7 days of drought. Protein carbonyls were detected after dinitrophenyl hydrazine (DNPH) labeling and quantification. Ct: control (well watered); WS: water stress (7 days). For each genotype/condition, 4 samples from two independent experiments were analyzed. Data correspond to molar carbonyl/protein ratios, means ± SD (*n* = 4). Samples significantly different (*p* < 0.05) are indicated by an asterisk (*).

**Figure 4:**
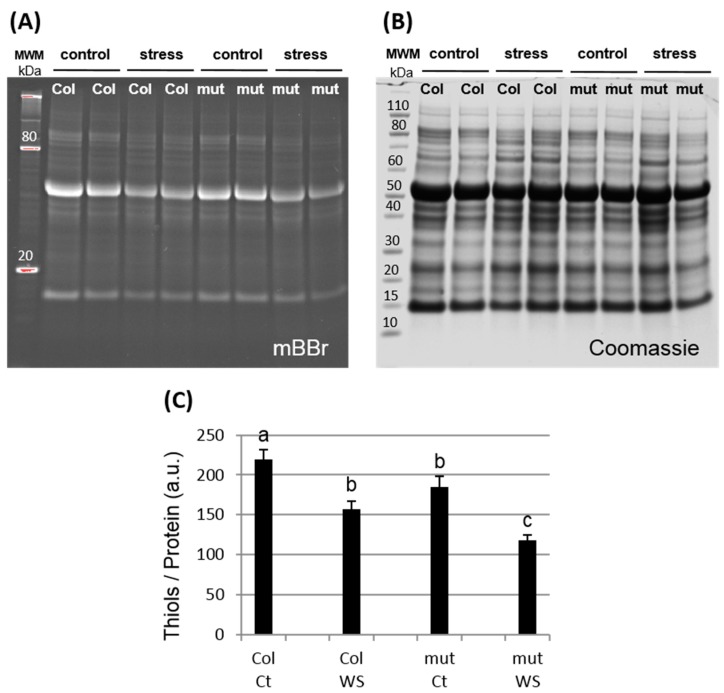
Protein thiols quantification in WT and *trxy1y2* mutant leaves after 7 days of drought. (**A**) mBBr fluorescent labelling of protein thiols after SDS-PAGE; (**B**) Coomassie staining of the same gel; (**C**) Thiol content relative to protein content. Ct: control (well watered); WS: water stress (7 days). MWM: molecular weight marker. a. u.: arbitrary unit. For each genotype/condition, 4 samples from two independent experiments were analyzed. Representative gels are shown in (**A**,**B**); quantification data were obtained using ImageLab software and means ± SD of fluorescent signal reported to Coomassie signal are shown in (**C**) (*n* = 4). Samples significantly different (*p* < 0.05) are indicated by different letters.

The extent of protein carbonylation, a stress-related PTM considered as a hallmark of protein oxidation was studied in the *trxy1y2* mutant [29]. After 7 days of dehydration, we found that protein carbonylation was higher in mutant plants compared to WT (Figure 3). The correlation between stress tolerance and protein oxidation level was even clearer when the global protein thiol content was quantified (Figure 4). In control conditions, proteins from mutant plant leaves had a lower thiol content of ca. 15% than WT, and this difference was even more pronounced in drought conditions (ca. 33%). These results suggested that loss of y-type TRX functions is accompanied by oxidative stress at the molecular level and that this effect might be a primary reason for the increased sensitivity of the corresponding mutants to water deficit.

### 3.3. The Ascorbate Pool is More Oxidized in the trxy1y2 Mutant During Drought Stress

**Figure 5:**
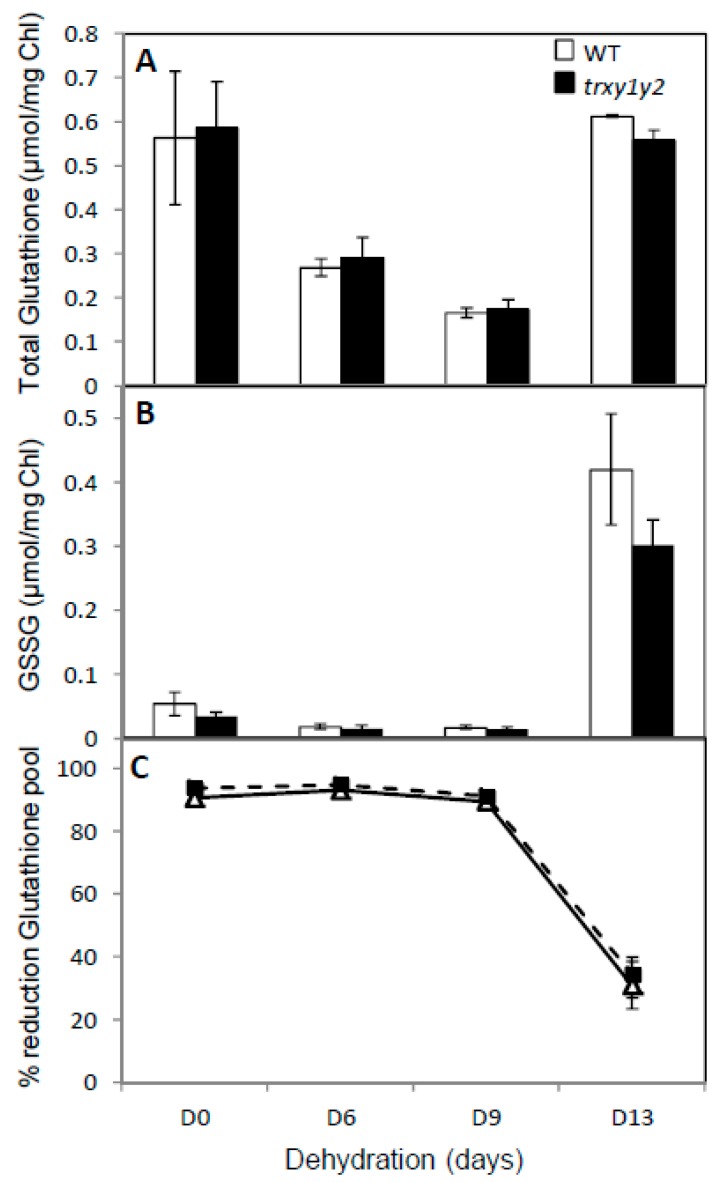
Effect of drought on leaf glutathione pool size and redox state. Glutathione pool after dehydration in WT (open column or triangle) and in *trxy1y2* mutant (dark column or square) leaves. (**A**) Total glutathione content; (**B**) GSSG content; (**C**) redox state of the glutathione pool. Data correspond to means ± SD (*n* = 4, from 2 independent experiments).

To further characterize the effect of the *trxy1* and *trxy2* mutations on leaf cellular redox homeostasis, we analyzed the redox state and the pool size of glutathione, a metabolite considered as a cellular redox buffer [30]. In both WT and mutant leaves 6 days without watering caused the total glutathione pool to decrease to about 50% of the control value. It was further affected after 9 days of drought but then recovered to initial levels (ca. 0.5 µmol/mg Chl) after 13 days of stress (Figure 5A). This effect was also observed for GSSG, the oxidized form of glutathione, which was strongly increased after 13 days of dehydration (Figure 5B), causing a drastic drop in the glutathione redox state from ca. 90% to ca. 30% (Figure 5C). Hence, the effects of drought on glutathione were comparable in the leaves of WT and mutant plants and could not explain their contrasting capacities to cope with water deprivation. 

**Figure 6:**
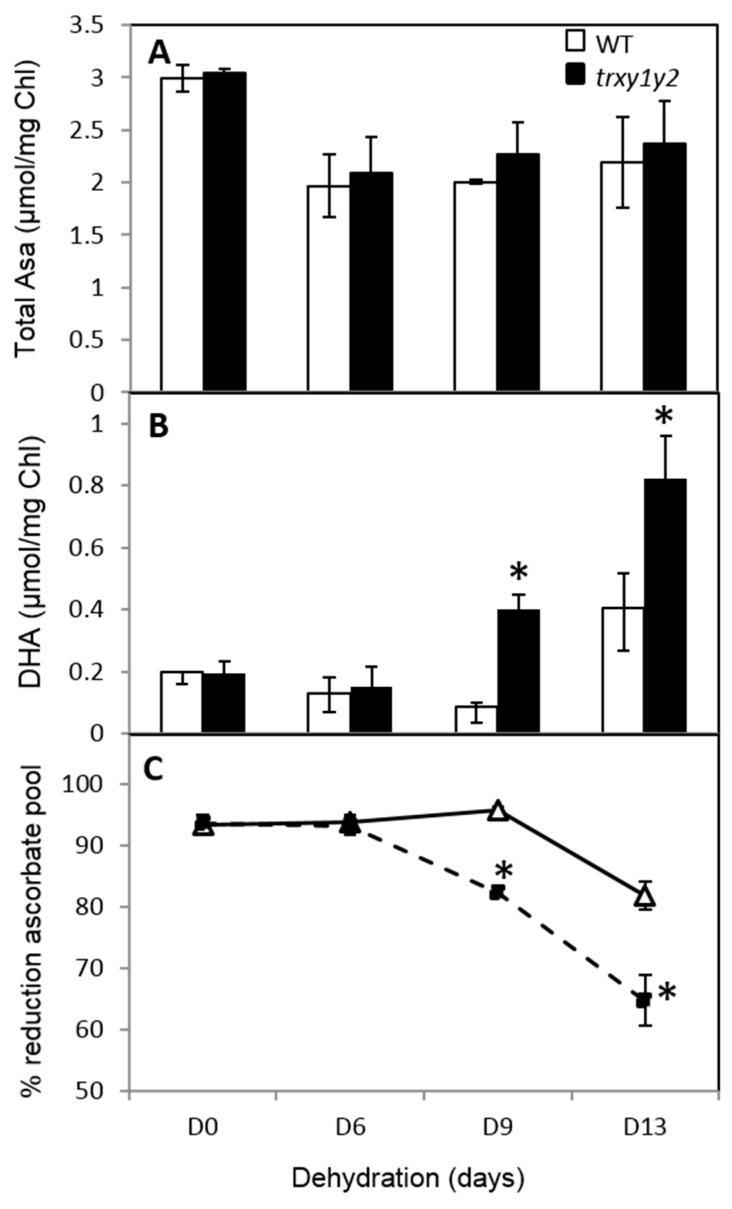
Effect of drought on the ascorbate pool size and redox state. Ascorbate (AsA) pool after dehydration in leaves from Col-0 (open bars or triangles) and in double *trxy1y2* mutant (dark bars or squares). (**A**) Total AsA content; (**B**) Oxidized AsA (DHA) content; (**C**) Redox state of the ascorbate pool. Data correspond to means ± SD (*n* = 4, from 2 independent experiments). Mutant samples significantly different from WT (*p* < 0.05) are indicated by an asterisk (*).

Ascorbate (AsA) is another low molecular weight antioxidant metabolite abundant in plant cells that plays an important role in tolerance to environmental constraints. It allows the alleviation of H_2_O_2_ accumulation through non-enzymatic and enzymatic pathways for scavenging ROS produced during stress [31,32,33]. Throughout the drought stress experiment, while the total pool size of AsA initially decreased (by ca. 30% at d6) and then remained mainly unchanged for both genotypes (Figure 6A), its oxidized form, DHA (dehydroascorbate) increased earlier and more markedly in mutant leaves compared to WT (Figure 6B). As a consequence, in WT leaves the reduction state of AsA remained high (ca. 95%) at d9 and decreased to ca. 85% at d13 whereas in mutant leaves it reached ca. 85% at d9 and dropped to ca. 65% at d13 (Figure 6C). Clearly, the leaf capacity to avoid AsA oxidation in response to drought seemed to be affected in the *trxy1y2* mutant in comparison to WT.

### 3.4. TRX y Can Increase MDHAR Activity in Leaf Crude Extracts

**Figure 7:**
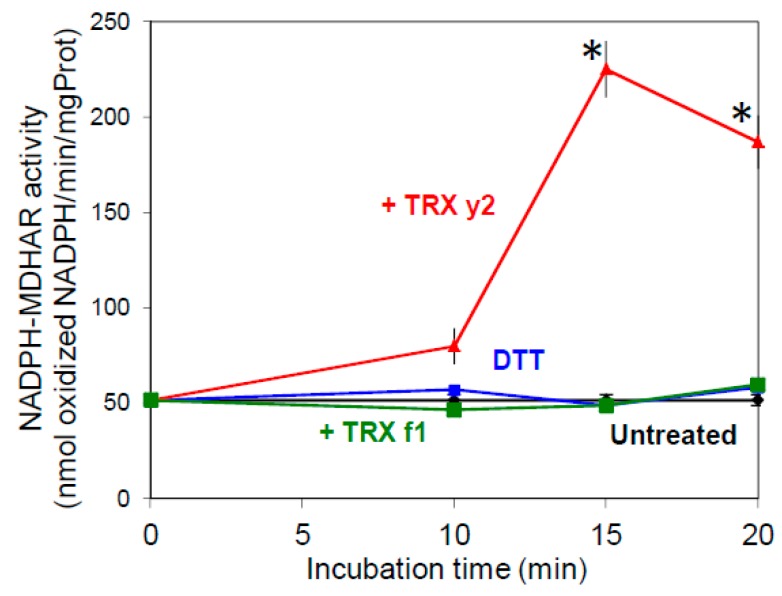
Effect of TRX on NADPH-dependent MDA reductase (MDHAR) activity in leaf extracts. Col-0 leaf protein extracts were incubated at room temperature in absence (black), or in presence of DTT, alone (blue), or with 20 μM TRX y2 (red), or TRX f1 (green) prior measuring NADPH-MDHAR activity. Data correspond to means ± SD (*n* = 4, from 2 independent experiments). Mutant samples significantly different from untreated (*p* < 0.05) are indicated by an asterisk (*).

Monodehydroascorbate (MDA) is the primary product of AsA oxidation occurring during H_2_O_2_ detoxification by ascorbate peroxidase which uses AsA as a reduced substrate. MDA, if not rapidly reduced, can also be further oxidized to DHA by a non-enzymatic reaction. In leaves, MDA reductase (MDHAR) (EC 1.6.5.4) plays a key role in AsA recycling by catalyzing MDA reduction using NAD(P)H as an electron donor [34]. Therefore, MDHAR plays an important role in the response of plants to oxidative stress by maintaining the intracellular AsA redox state mainly in its reduced state. Past work in the laboratory unraveled that MDHAR was a potential target of TRX y and we found that adding reduced TRX y1 (a TRX isoform expressed in non-photosynthetic tissues) to crude root extracts strongly increased MDHAR activity [19]. Recently, we also showed that leaf extractable MDHAR activity was markedly increased in the photorespiratory *cat2* mutant, deficient in the major catalase isoform of Arabidopsis leaves [35], suggesting redox sensitivity of this enzyme in leaves too [36]. To test this hypothesis and a possible regulatory role of TRXs y towards MDHAR in leaves, we incubated WT leaf extracts in the presence of purified plastidial TRXs. While pre-incubation with the chemical reductant DTT alone or in presence of TRX f1 did not change NADPH-dependent MDHAR activity (which is mainly attributable to the organellar isoform), this activity was increased 4-fold after a 15 min treatment in the presence of reduced TRX y2, the y-type TRX isoform expressed in Arabidopsis leaves (Figure 7). Interestingly, the same reducing treatments showed no effect on NADH-dependent activity attributable to cytosolic isoforms (Figure S1). The incubation in oxidizing conditions, using the strong oxidant trans-4,5-Dihydroxy-1,2-dithiane (DTTox), did not change the NADPH-dependent activity in leaf extracts suggesting a spontaneous complete oxidation of the enzyme upon sample preparation (data not shown). Thus, taken together, these results strongly suggested a role for TRX y in the regulation of NADPH-dependent MDHAR activity in leaves.

### 3.5. In Vitro Validation of a TRX y-Specific Regulation of AtMDHAR6 Activity

**Figure 8:**
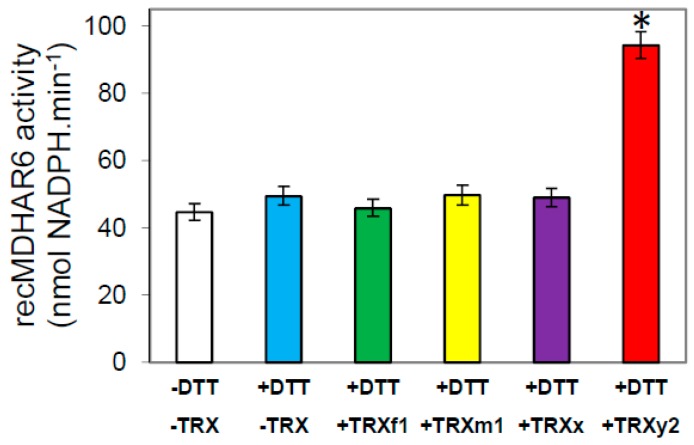
Effect of reducing treatments on AtMDHAR6 activity. A sample of purified recombinant AtMDHAR6 enzyme was incubated for 15 min at room temperature prior to measuring its NADPH-dependent activity. Incubation was in absence of DTT (white bar), or in presence of DTT, alone (blue bar), or with TRX: f1 (green bar), or m1 (yellow bar), or x (purple bar), or y2 (red bar). Data correspond to means ± SD (*n* = 4, from 2 independent experiments). The treatment significantly increasing NADPH-MDHAR activity (comparison with untreated) (*p* < 0.05) is indicated by an asterisk (*).

In Arabidopsis, 6 MDHAR isoforms were found and MDHAR6 was shown to be plastid-targeted and to use specifically NADPH as reducing cofactor for catalysis [37]. Therefore, we cloned the cDNA of MDHAR6 from Arabidopsis (AtMDHAR6) into an *E. coli* expression plasmid allowing its production in its mature form with a strep-tag at its C-terminal end. The corresponding recombinant protein was purified to homogeneity (Figure S2) and its activity was tested for sensitivity to redox treatments. In vitro, recombinant AtMDHAR6 activity was increased more than two-fold by reduced TRX y2, while the other TRXs tested (of the f, m, or x types) had no effect on the activity of this enzyme (Figure 8).

## 4. Discussion and Conclusions

### 4.1. TRX y Depletion Leads to a Higher Sensitivity of Arabidopsis to Drought Stress

The importance of oxidative stress and the role of ROS in local and systemic signaling in plants in response to drought has been largely documented (see for reviews [38,39,40]) and the induction of oxidative stress occurring as a drought effect is now widely accepted. This implies that a limited availability of water favors an imbalance between the production of ROS and their elimination. This leads to an increase in ROS levels such as H_2_O_2_ and singlet oxygen (^1^O_2_) during drought [40], even though the probability of ^1^O_2_ production may be low [22]. Besides, previous biochemical studies on plastidial TRXs have revealed that y-type TRXs are preferential substrates for antioxidant enzymes such as Peroxiredoxin (PRX) Q [5,15], Glutathione Peroxidase (GPX1) [6], and Methionine Sulfoxide Reductase (MSR B2) [7]. Furthermore, the involvement of TRX y2 in the maintenance of leaf MSR capacity was demonstrated in vivo and the corresponding mutants both showed altered capacities to grow in high light/long day conditions [17]. Thus, we wondered whether y-type TRXs could be functionally important in the tolerance of Arabidopsis to other pro-oxidative stress conditions such as drought. We first challenged Arabidopsis plants lacking TRX y1 or y2 with water deficiency and found that they had an altered tolerance as compared with wild-type (WT) plants.

Then, we obtained a *trxy1y2* double mutant in which we performed a comparative study of water stress physiological effects and antioxidant responses relative to WT plants. We found that in control growth conditions, while showing no phenotype, the mutant had a lower leaf osmotic potential (−11.25 bar) compared to WT (−9.8 bar, data not shown). The *trxy1y2* mutant exhibited an enhanced sensitivity to drought correlating with a reduced capacity to keep high leaf water content (Figure 2).

### 4.2. TRX y is Necessary for Antioxidant Responses During Drought Stress

At the molecular level, in stress conditions, this importance of TRX y was underscored by increased levels of protein carbonylation and thiol oxidation in the *trxy1y2* mutant in comparison with WT (Figure 3 and Figure 4). Thus, in mutant leaves the protein global redox status was modified towards oxidation. Moreover, the antioxidant metabolites ascorbate and glutathione, often used as biochemical markers of the cellular redox state, were quite strongly affected in our mild drought conditions. However, in both lines (WT and *trxy1y2* mutant), the glutathione content and redox state, as well as the total ascorbate content followed the same progressive decreasing trend in response to water deficiency (Figure 5 and Figure 6). It is worth mentioning that past studies carried out on how plant antioxidant systems respond to drought revealed a high degree of complexity and a large diversity between plant species, making generalizations difficult [22]. A transcriptomic study on Arabidopsis plants exposed to drought stress drew similar conclusions [41]. The major difference observed in our comparative study was in the redox state of the ascorbate (AsA) pool. Indeed, under water deprivation the *trxy1y2* mutant showed an AsA pool that oxidized more rapidly and markedly than in the WT (Figure 6). Thus, one reason why the TRX mutant is more sensitive to drought could be its limited capacity to regenerate the AsA pool and maintain its antioxidant capacity. Moreover, chloroplast AsA Peroxidases (APXs) have been shown to be highly sensitive to oxidative inactivation in the absence of reduced AsA [42]. Thus, the drought stress sensitivity of the *trxy1y2* mutant could be linked to its impaired capacity to regenerate AsA from the radical MDA, with possible consequences for the antioxidant function of APXs.

### 4.3. TRX y Controls the Reduction of the Ascorbate Pool in Redox Regulating the Plastidial MDHAR

MDA reductase (MDHAR) is known to play an important role in the response of plants to oxidative stress by maintaining intracellular AsA redox state mainly in its reduced state. Past studies have evidenced a direct relationship between stress tolerance and MDHAR leaf activity [43]. Furthermore, the activity of MDHAR was found to be increased by diverse stresses including drought and salt stress [44,45,46,47,48]. Other studies suggest that MDHAR plays a key role in the regeneration of AsA and in tolerance to oxidative stress in plants [20,49]. Consistent with this notion, overexpression of Arabidopsis MDHAR1 (MDAR1) in tobacco plants enhanced their tolerance to ozone and increased their photosynthetic activity under salt stress [50]. In agreement with the possibility of a functional link between y-type TRXs and MDHAR in Arabidopsis, we previously identified the plastid MDHAR isoform (MDHAR6) as a putative target of TRX y2 [19]. We showed that NADPH-dependent MDHAR activity was enhanced in Arabidopsis root extracts by incubation with reduced TRX y1 whose gene is mostly expressed in non-photosynthetic tissues. In the present study, we have reported that NADPH-MDHAR activity can be also strongly increased in leaf extracts by reduced TRX y2 (Figure 7), while NADH-dependent MDHAR activity cannot and TRX f has no effect on leaf extractible MDHAR activity. Furthermore, we were able to validate in vitro a direct regulation of recombinant plastidial NADPH-MDHAR6 activity by TRX y2 (Figure 8). Since the other plastidial TRXs we tested had no effect, it seems that regulation of NAPH-MDHAR is specific to the y type. It is worth mentioning that the inefficiency of the other TRXs to activate MDHAR cannot be linked to their lower reducing capacity since TRXs y have a less negative redox potential [5,6,15]. Instead, steric and electrostatic complementarities between TRX y and its target enzyme might be determinants of specificity. Interestingly, multiple alignments of higher plant MDHAR primary sequences indicate that 3 out of 4 Cys are conserved in plastid isoforms [19]. Future work will allow identifying the cysteines involved in the activation process of NADPH-MDHAR by TRXs y.

### 4.4. Physiological Relevance of TRX-Dependent MDHAR Regulation in Chloroplasts

The present work reveals a new function for y-type TRXs and underlines their role as major thiol-based antioxidant proteins in plastids. This newly validated regulation might be physiologically relevant not only under water deprivation but also in other stress conditions where MDHAR capacity has been correlated with plant tolerance, as well as under normal growth conditions when a strong capacity for the reduction of the AsA pool is required, for example at sunrise. Indeed, it has been shown that during the night, the AsA pool size can decrease [51], and that the biosynthesis of AsA requires the activity of the photosynthetic electron transport and is therefore light-dependent [52]. In addition, AsA synthesis and regeneration can be influenced by the quality and the amount of light [53]. Because TRX y2 reduction is directly linked to the photosynthetic electron transport chain by ferredoxin/TRX reductase [9,15], the TRX-dependent regulation of MDHAR may allow its activity to be coordinated with the production of its reductant, NADPH, in chloroplasts exposed to changing conditions such as irradiance.

## Supplementary Materials

The following are available online at http://www.mdpi.com/2076-3921/7/12/183/s1, Figure S1. Effect of TRX treatments on NADH-dependent MDHAR activity in leaf extracts. Figure S2. Purification of recMDHAR6. Table S1. List of primers used for cloning AtMDHAR6 cDNA in pGENI expression plasmid.
