# Supplementary material for: Redox Regulation of Monodehydroascorbate Reductase by Thioredoxin y in Plastids Revealed in the Context of Water Stress
Source: Antioxidants (Basel). 2018 Dec 6;7(12):183. doi: 10.3390/antiox7120183 (PMC6316508; doi:10.3390/antiox7120183)
Supplement: Supplementary file 1 [file antioxidants-07-00183-s001.pdf]

antioxidants

SupplementaryMaterials

Table S1. List of primers used for cloning AtMDHAR6 cDNA in pGENI expression plasmid.

MDHAR6-up-Nco	AAACCATGGCATTTTCTCCGATCGGTTCTAG
MDHAR6-down-Xho	GGAAACTTCATGGAGTTTTTTTAACTCGAGAAA
pET-seq-up	CTCTAGAAATAATTTTGTTTAACTTTAAGAAGG
pET-seq-down	GCTGAGCAATAACTAGCATAA

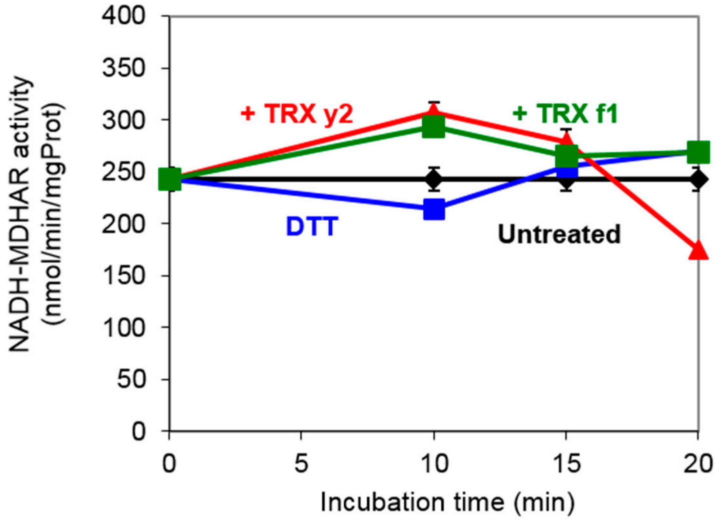

Figure S1. Effect of TRX treatments on NADH-dependent MDHAR activity in leaf extracts. Col-0 leaf protein extracts were incubated at room temperature in absence (black), or in presence of 0.5 mM DTT, alone (blue), or with 20 µM TRX y2 (red), or TRX f1 (green) prior to measuring NADH-MDHAR activity. Data correspond to means ± SD (*n* = 4, from 2 independent experiments).

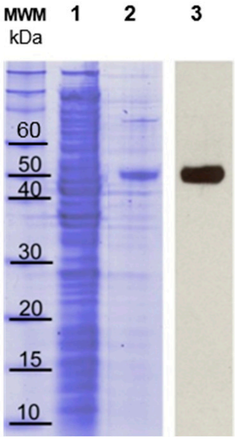

Figure S2. Purification of recMDHAR6. Protein sample analysis by SDS-PAGE and Coomassie staining : (**1**) Crude extract of *E. coli* cells producing recMDHAR6 (50 µg of soluble crude extract), (**2**) Purified recMDHAR6 (3 µg). Immunodetection of purified recMDHAR6 (**3**) by western blot using anti-strep-tag antibody as described in ref. [9].
